# Plant emotion revisited: toward a new conceptual framework

**DOI:** 10.1007/s00709-026-02169-y

**Published:** 2026-02-11

**Authors:** Katia Forsman, Jari Jussila

**Affiliations:** 1 Salo, Finland; 2 HAMK Design Factory, Häme University of Applied Sciences, Vankanlähde 9, Hämeenlinna, 13100 Finland

**Keywords:** Plant emotion, Emotus plantarum, Core affect, Plant signaling, Plant neurobiology, Plant sentience, Plant consciousness

## Abstract

Traditional paradigms in basic emotion research, largely grounded in animal and human psychology, are problematic when extended to plants due to fundamental biological and neurophysiological differences. This paper criticizes the anthropocentric tendency to equate plant responses with human emotional states, emphasizing that plants, lacking a central nervous system and brain, cannot experience feelings in the human sense. Instead, we propose a novel conceptual framework: emotus plantarum. This term defines a plant-specific signaling system that fulfills a role functionally analogous to emotions in animals—coordinating adaptive responses to environmental stimuli. Unlike cognitive models that presuppose centralized information processing, emotus plantarum reframes abilities such as learning, memory, anticipation, and decision-making as emergent, decentralized properties of plant physiology that serve emotion system functions. These processes modulate plant behavior, such as tropisms, nastic movements, and chemical signaling enabling flexible, context-dependent reactions that enhance survival and fitness. This framework shift allows for a more biologically appropriate interpretation of plant behavior, bypassing the need to force-fit plant responses into anthropomorphic categories. By repositioning these attributes within an emotion system framework unique to plants, the concept of emotus plantarum offers a more nuanced approach to understanding plant-environment interactions. Ultimately, this perspective promotes a deeper integration of plant-specific biology into the study of adaptive behavior, opening new avenues for research in plant signaling, communication, and responsiveness.

## Introduction

Plant emotions have been studied for a long time, but without major breakthroughs. In this study, we examine the essential research in the field. Previous research on plant emotions has often been framed using emotion research paradigms about basic emotions, tending to translate plant behaviors into the language of human basic emotions (cf. Cho et al. 2015; Chung et al. 2017; Wase et al. 2023). We find this paradigm problematic because research indicates that plants, lacking a brain, do not experience feelings in the way humans do (Struik 2023).

Some researchers of plant intelligence or sentience connect plants to sentience and affect. For instance, Gagliano (2017, p. 1) writes regarding plants: “processes like learning, memory, anticipation, awareness, decision-making and more, which are, broadly speaking, attributes of what we call, the mind.” Calvo and Lawrence (2022) argue that plants might have subjective experiences, not human emotions or feelings of pain in the animal sense, but internal states shaped by prediction, mismatch and adaptation.

These qualities can be equally considered attributes of emotion (cf. Barlow 2010). Emotion changes focus of perception, attention and memory and effects to decision making (Brosch et al. 2013; Gannouni et al. 2021). We propose that plant qualities and abilities, such as learning, memory, anticipation, and decision-making, which have been widely considered attributes of the mind, may instead be attributes of emotion. Plants possess the functions required for the existence of bodily emotion, but not the psychological qualities of the mind, as they lack a brain. Because automatic emotional processes are possible without awareness (Delplanque and Sander 2021), we propose plants are capable for them.

According to Gagliano (2017) plants “feel” and “experience”. We argue that plants, lacking a brain, do not “feel” or “experience”. However, we propose that plants possess a plant-specific signaling system, closely associated with their movement and adaptive responses, that may serve a functional role analogous to an emotional system in animals. We consider linking research on plants emotions to biopsychism unsuitable, as current studies have not demonstrated that plants are sentient (Mallatt et al. 2021, 2023; Laist 2024).

## Methods

A scoping study (Levac et al. 2010) was selected as the initial method to investigate essential research on plant emotions. The literature search was conducted in two phases. In the first phase, the Scopus database was selected as the primary source for the literature search due to its extensive coverage of peer-reviewed journals across multiple scientific disciplines. The initial search was limited to plant emotions and plant feelings, using the search terms “plant emotion*” and “plant feel*” to search within Article title, Abstract and Keywords in all subject areas and document types.

Due to a limited amount of search results, the scoping study was expanded in the second phase to cover the most cited research on the topic in Google Scholar. The expanded search covered both research published in scientific journals and also grey literature (Levac et al. 2010). The second phase identified as a major debate in the literature the claims and counterclaims about plant consciousness (Mallatt et al. 2021, 2023; Tompkins and Bird 1973; Laist 2024; Backster 2023) and a missing definition of a plant-specific emotion system. The results first introduce and summarize the research identified in the scoping study and then proceed to define a plant-specific emotion system. Following this, the discussion revisits claims and counter claims of plant consciousness from the perspective of the newly proposed emotion-system framework.

## Results

Investigating the Scopus database, the search string “plant emotion*” yielded seven results and the search string “plant feel*” 20 results. From these initial search results, three articles were excluded, which were short magazine articles or editorials, further three articles were excluded as they introduced technical solutions but did not especially contribute to understanding of plant emotions, and finally seven articles were excluded, which were not at all related to plant emotions. Overall, 11 articles were evaluated based on the abstract to address the topic of plant emotions. The included articles and their key concepts are next summarized in Table 1.

**Table 1:** Summary of titles and authors, publication, and key concepts in the discovered articles

Title and author(s)	Publication	Key concepts
How do plants feel the heat and survive? (Guihur et al. 2022a)	Trends in biochemical sciences	heat, heat sensing, heat signaling
How do humans and plants feel the heat? (Guihur et al. 2022b)	Trends in plant science	heat, heat sensing, heat signaling
Anesthetics and plants: no pain, no brain, and therefore no consciousness (Draguhn et al. 2021)	Protoplasma	anesthetics, sleep, ion channels, perception, cognition
Do plants feel pain? (Hamilton and McBrayer 2020)	Disputatio: International Journal of Philosophy	pain, phenomenal consciousness, qualia
Photomorphogenesis: plants feel blue in the shade (Franklin 2016)	Current Biology	escape responses, shade avoidance, light signals
How do plants feel the heat? (Mittler et al. 2012)	Trends in biochemical sciences	heat, heat stress, heat stress response
Ion homeostasis: plants feel better with proper control (Mueller-Roeber and Dreyer 2007)	EMBO reports	ion homeostasis, plants feel
Communicating with Plants: The Dream, the Science, the Everyday Reality (Laist 2024)	Environmental Humanities	plant communication, plant agency
An IoT-Enabled Smart Gardening System as a Novel Approach to Environmental Education (Wase et al. 2023)	IEEE Renewable Energy and Sustainable E-Mobility Conference	plant emotions, internet of things, augmented reality
Study of plant emotion using music and motion detection in Internet of Things (Chung et al. 2017)	International Conference on Ubiquitous and Future Networks	plant emotions, internet of things, music
People’s Emotional Responses to a Plant’s Emotional Expression (Cho et al. 2015)	International conference on tangible, embedded, and embodied interaction	plant emotions, emotional expression

The body of research outlined in Table 1 converges on the idea that plants possess highly sophisticated mechanisms for sensing and responding to their environment, but these mechanisms are fundamentally different from animal emotions or consciousness. Studies show that plants can detect and react to various stimuli, such as heat, light, and changes in their internal or external environment through intricate molecular, electrical, and physiological signaling systems (Mittler et al. 2012; Franklin 2016; Guihur et al. 2022a, b). These responses are adaptive and coordinated, demonstrating that plants are active participants in their own survival rather than passive organisms. However, the consensus across these works is that, while plant behaviors may appear “emotion-like,” they do not constitute emotions or feelings in the human or animal sense. Additionally, some research explores how humans interpret plant behaviors, often projecting emotional states onto plants or using technology to monitor plant “well-being” (Cho et al. 2015; Chung et al. 2017; Wase et al. 2023).These perspectives highlight the anthropomorphic tendencies in human-plant relationships, but do not provide evidence that plants themselves possess emotions.

In terms of scholarly impact of these articles, two articles related to plants feeling heat by (Mittler et al. 2012) and (Guihur et al. 2022a) were relatively highly cited, but the other articles had received only small number of citations. The second phase of the scoping study explored the most cited research on the topic in Google Scholar, beginning from backward citations of the articles identified in the first phase (cf. Laist 2024) and then of those cited literature. The top 20 most cited research is illustrated in Table 2, with only one article overlapping with the Scopus search results.

**Table 2:** Top 20 cited research on plants emotions, publication, key concept(s), and number of citations

Title and author(s)	Publication	Key concept(s)	Cit.
Plant-thinking: A philosophy of vegetal life (Marder 2013)	Columbia University Press (Book)	plant thinking, plant philosophy	1410
How do plants feel the heat? (Mittler et al. 2012)	Trends in Biochemical Sciences	heat, heat stress, heat stress response (HSR)	1197
The secret life of plants (Tompkins and Bird 1973)	Harper & Row (Book)	plant consciousness, plant feelings, plant emotions, plant thinking	807
Aspects of plant intelligence (Trewavas 2003)	Annals of Botany	plant intelligence	707
What a Plant Knows: A Field Guide to the Senses: Updated and Expanded Edition (Chamovitz 2020)	Scientific American/FSG (Book)	plant perception	597
Plant neurobiology: an integrated view of plant signaling (Brenner et al. 2006)	Trends in Plant Science	plant neurobiology, plant behavior	573
Brilliant green: the surprising history and science of plant intelligence (Mancuso and Viola 2015)	Island Press (Book)	plant intelligence	476
*The life of plants: A metaphysics of mixture* (Coccia 2019)	John Wiley & Sons (Book)	plant philosophy	376
Through vegetal being: Two philosophical perspectives (Irigaray and Marder 2016)	Columbia University Press (Book)	plant philosophy	368
The nervous mechanism of plants (Bose 1926)	Longmans, Green & Co (Book)	plant electrophysiology	282
Plants: Adaptive behavior, root-brains, and minimal cognition (Calvo Garzón and Keijzer 2011)	Adaptive Behavior	plant intelligence, plant neurobiology	259
*The philosopher’s plant: an intellectual herbarium* (Marder 2014)	Columbia University Press (Book)	plant philosophy	258
The foundations of plant intelligence (Trewavas 2017)	Interface Focus	plant intelligence	197
Plant intelligence (Trewavas 2005)	Naturwissenschaften	plant intelligence	168
Plant intentionality and the phenomenological framework of plant intelligence. (Marder 2012)	Plant signaling & behavior	plant philosophy	160
The revolutionary genius of plants: a new understanding of plant intelligence and behavior (Mancuso 2018)	Simon and Schuster (Book)	plant intelligence, plant behavior	155
Consciousness and cognition in plants (Segundo-Ortin and Calvo 2022)	WIREs Cognitive Science	plant cognition, plant consciousness, plant neurobiology	140
Métamorphoses (Cocchia 2020)	Éditions Rivages (Book)	plant philosophy	130
Planta sapiens: unmasking plant intelligence (Calvo and Lawrence 2022)	Hachette UK (Book)	plant intelligence, plant cognition	127
Debunking a myth: plant consciousness (Mallatt et al. 2021)	Protoplasma	plant consciousness, plant electrophysiology	118

A significant portion of the most-cited literature explores the philosophical dimensions of plant life, challenging traditional human-centered views. Authors such as Marder (2012, 2013, 2014), Irigaray (2016), Cocchia (2019; 2020), and Mallatt et al. (2021; 2023) argue for a rethinking of plant subjectivity, ethics, and the metaphysical status of plants. These works do not claim that plants have emotions in the human sense, but instead invite us to reconsider plants as active, sentient participants in the world, deserving of ethical consideration and a place in philosophical discourse.

Another major theme is the argument that plants exhibit forms of intelligence and cognition, albeit without a central nervous system. Research by Trewavas (2003, 2005, 2017, 2021), Trewavas and Baluška (2011), Mancuso (2015; 2018), Calvo (2011; 2022), and others presents evidence that plants can sense their environment, communicate chemically, adapt their behavior, and even display memory and decision-making. These studies redefine intelligence as adaptive behavior and propose that plants process information in decentralized ways, sometimes described as “root-brains” (Mancuso 2018; Baluška and Mancuso 2021) referring back to Darwin’s (1898) ideas, or minimal cognition (Calvo Garzón and Keijzer 2011; Segundo-Ortin and Calvo 2022).

Several articles focus on the biological and physiological mechanisms underlying plant perception. These works provide detailed scientific foundations for how plants sense and respond to environmental stimuli such as heat, light, and touch. They show that plants are not passive but possess sophisticated sensory systems, including electrical signaling and heat-stress responses, which allow them to actively detect and adapt to their surroundings (Bose 1926; Brenner et al. 2006; Mittler et al. 2012; Chamovitz 2020).

Some influential books, such as Tompkins and Bird (1973), basing their arguments on the work of Cleve Backster (Laist 2024; see also Backster 2023), have popularized the idea of plant emotions and consciousness, often based on anecdotal or pseudoscientific claims. While these have contributed to public interest, later research has critically examined and often refuted the notion that plants possess emotions or consciousness in a literal sense, emphasizing the need for scientific rigor and clear definitions (Mallatt et al. 2021, 2023; Laist 2024).

Based on the current evidence, plants do not possess neurobiological structures analogous to those found in animals. Consequently, the concept of ‘plant neurobiology’ may be misleading, as some interpretations of plant signaling and behavior rely on analogies that do not accurately reflect underlying mechanisms. Although neurotransmitters have been detected in plants (Brenner et al. 2006), their presence does not indicate that plants possess a nervous system. Instead, these signaling molecules may participate in what can be conceptually described as a plant ‘emotion system’, mediating responsiveness and coordinated behaviors.

From our perspective experiments of Bose (1926) more accurately indicate plants having an emotion system rather than nervous system. Because plants do not have anatomically defined nervous system, it is reasonable to assume nervous system like functioning in plants indicates rather a plant emotion system than a nervous system. Plant emotion system requires such electrical functioning through phloem as Bose (1926) proved through his experiments.

Regarding the literature included in this review, it appears that several researchers, in their respective works, approach similar conclusions from different directions. These conclusions can be interpreted as components or building blocks for the development of a theory of *emotus plantarum*, provided that the underlying logic is slightly reframed and the relevant concepts are clarified.

For instance, Mancuso (2018) comes, in our view, close to the idea of *emotus plantarum* by describing plant decision-making processes that are based on a form of rationality distinct from brain-based cognition, yet not irrational. He interprets this capacity as an outcome of evolutionary processes shared by plants and humans. Although this perspective aligns in several respects with the concept of plant emotion or *emotus plantarum*, Mancuso does not explicitly characterize these phenomena as emotional. Similarly, Calvo and Lawrence (2022) introduce the concept of *planta sapiens*, which in our view bears conceptual similarities to *emotus plantarum*, but differs in scope, content, and definition. None of the reviewed studies employ the concept of (bodily) emotion, nor do they explicitly specify the emotional nature of the plant phenomena they describe. Marder (2012, 2013, 2014) focuses on the notion of plant bodily thinking, which we interpret as potentially referring to a plant’s capacity to process bodily emotion through mechanisms that could be described as unconscious cognition. However, Marder does not frame plant thinking in these terms, nor does the concept of *emotus plantarum* appear in his philosophical work.

## Definition of emotus plantarum

- A. Introduction of the New Paradigm

From our perspective, many plant behaviors previously interpreted as indicators of intelligence may instead reflect ‘emotus plantarum,’ a plant-specific emotion system. We propose a new research paradigm for studying plant emotions and associated behaviors, informed by recent advances in the field of emotion research. According to Barrett (2006), contemporary emotion research is grounded in the concept of neurophysiological ‘core affect,’ which serves as a fundamental substrate of emotional experience, providing ‘core information,’ ‘core knowledge,’ and ‘core experience,’ rather than focusing solely on discrete basic emotions such as fear or anger. We propose basic human emotions are too abstract for plants. Any emotion observed in plants is hypothesized to be grounded in measurable physiological, biochemical, or electrical mechanisms. There is ongoing debate among researchers regarding whether basic emotions, such as fear and anger, represent natural, biologically hard-wired phenomena or are shaped primarily by cultural and contextual factors (Barrett 2006).

According to Russell (2009), ‘core affect’ refers to a neurophysiological state that is for humans consciously accessible as a simple feeling, characterized by two primary dimensions: valence (pleasantness vs. unpleasantness) and arousal (activation vs. deactivation). Core affect is more neurophysiological and bodily in nature, closely connected to biology, whereas basic emotions are interpretations by the brain of bodily sensations shaped through cultural context, making them more complex psychological experiences than core affect. Through core affect emotion research can reach beyond the use of basic human emotions as scientific tools for emotion research (Russell 2009), and thus is more suitable for research on emotus plantarum. We propose plants have emotion similar to “core affect” that however may not have connection to consciousness in the absence of a brain and thus may not be called “core affect”.

- B. Principles of Core Affect

The emerging paradigm in emotion research has greater conceptual compatibility with studies addressing plant-related emotional processes and qualities (Barrett 2006, 2012). “Core affect” is non cognitive, does not require thought, arises automatically, is universal among several species and is like a “barometer” of one’s inner state (Thomson and Coates 2021). Plants are open thermodynamic systems that acquire information through biochemical and biophysical signaling processes that depend on energy exchange with the environment. Like all living systems, plants maintain internal order by importing low-entropy energy and matter and exporting entropy to their surroundings. While these processes enable adaptive responses to environmental stimuli, they should not be equated directly with human emotional systems. Accordingly, plants can be understood as engaging in information exchange with their environment through mechanisms conceptualized as emotus plantarum. Researchers in fields such as plant neurobiology, ecology, and philosophy of biology have suggested that plants may be regarded as exhibiting forms of agency (Callaway and Walker 1997; Marder 2012, 2013; Baluška and Mancuso 2021; Novoplansky et al. 2024; Rutten et al. 2025). We introduce a new concept of emotus plantarum that we hypothesize to operate as mechanisms of agency, exhibiting parallels to the functional role of authentic emotions in humans. This form of emotion represents a core functional property that confers a type of agency, even though plants lack a ‘self,’ ‘ego,’ or ‘soul’ as understood in human contexts.

- C. Definition of Emotus Plantarum

Darwin sought to investigate the biological underpinnings of behavior through the study of plant movements. He proposed that plants contain a brain-like structure that serves to integrate and regulate their sensory and responsive capacities (Darwin 1898). Some researches connected those sensitivities even to human thought (Whippo and Hangarter 2006). We propose those sensitivities are qualities of emotional system of the plant that is intertwined with plant movement system. The emotion system of the plant can be compared to emotion system of human by understanding human emotion through core affect (Barrett 2006; Russell 2009; Thomson and Coates 2021). Existence of core affect or emotus plantarum can be recognized through measuring movements of electrical signals technically. This perspective enables us to study how emotion affects plant movements or human behavior.

The point of view of Darwin in research of animal emotions is relatively anthropomorphic (LeDoux 2021). Darwin studied emotions from the point of view of basic emotions also regarding animals. Perhaps that is why Darwin did not connect his research on sensitive adaptive plant movements (Whippo and Hangarter 2009) directly with his studies on human and animal emotions. Studying emotions and behavior from the point of view of core affect is essential to broaden understanding evolution of behavior of organisms from renewed non anthropomorphic point of view.

From our point of view researchers of plant neurobiology are studying plant behavior from the point of view of sensitive brain- like organ in plant instead of sensitive emotion system in plant. They have for instance supposed that plant has brain -like organ in roots (Mallatt et al. 2021). However, their studies have not led to a breakthrough. According to our research, one reason for it might be that plants have plant emotion system typical to species of plant instead of brain-like organ. Plant signaling system typical to plant indicates such emotion system of plant that has similarities of emotion system of human, if emotions are studied from neurophysiological point of view instead of psychological point of view. Understanding this difference in logic regarding research on qualities of plants changes interpretation of several phenomena in plant behavior and how it can be connected to phenomena regarding humans.

- D. Emotus Plantarum and Physiological Systems

In plants electrical currents can be found that function as “core information” for plants. Similar electrical current have been measured in studies regarding bodily human emotions (Backster 2023) that also function as base of “core experience” and “core knowledge” for humans. Plant emotion might function through phloem, plasmodesmata and gap junctions in plant (cf. Koenig and Hoffmann-Benning 2020; Yatta 2023). We propose that plant emotion is a distinct concept from emotus plantarum as plant cannot in the absence of a brain form “core experience” or “core knowledge” about “core information” that we infer to be functions to emotion of plant.

Plant intelligence could be, according to our point of view, explained as an ability of the plant to process emotus plantarum into intelligent behavior. According to this viewpoint plants are not sentient the way humans are having brain-based consciousness. However this viewpoint does not exclude possibility that plants would have some kind of brainless “awareness” (Trewavas 2021) as a result of processing emotus plantarum that is transporting information. In our view if plants have “awareness”, we suppose it is some plant specific phenomena different from human awareness or consciousness. Our aim is to demonstrate that plants have plant specific emotion system.

Plant neurobiologists study plant behavior that is according to them “coordinated by some form, of integrated signaling, communication and response system” (Brenner et al. 2006). Current research does not, however, define what this system exactly is. We argue that this system is the emotion system of the plant, which has similarities with emotion system of human, not basic emotion system of human, but bodily emotion and emotion system based on new paradigm in emotion research (cf. Barrett 2006; Wells 2019).

## Discussion

Some researchers consider plants as sentient or having consciousness. Other researchers have critical point of view regarding plant consciousness and plant sentience (Mallatt et al. 2021, 2023). Some researchers have also concluded that plants do not have ability to feel (Struik 2023). According to them plants detect and adapt, but they do not consider that such behavior can be emotional without the ability to feel. Although we cannot share feelings with plants (Struik 2023), sharing sensation based bodily emotions with plants can be possible in our view. For instance, according to previous research different plant colors have been found to affect human emotions in different ways (Sadek et al. 2013).

In previous research, Mallatt et al. (2021; 2023) have addressed the 12 core claims about plant consciousness and provided their counterarguments for these claims. Responding to these claims and counterarguments we introduce a new scenario and paradigm for research on qualities of plants (Table 3). We argue that there is potential for new discoveries in both the claims for plant consciousness and in their counterarguments, and that emotus plantarum can offer new insights to this debate. The new scenario aims to address unanswered questions in the debate on the qualities of plants.

**Table 3:** Claims and counter claims for plant consciousness including a new interpretation based on the emotus plantarum concept

Claims for plant consciousness	Counter claims provided by Mallatt et al. 2021	Interpretation of claims based on the Emotus Plantarum
1. Each living cell is conscious	1. Cells sense and respond through adaptive plasticity, with complex actions explainable by non-conscious mechanisms rather than cellular consciousness.	**1. Conscious Cells and Emotus Plantarum**: Plant emotion theory does not require living cells to be conscious. Unconscious emotional processes are possible without awareness.
2. Consciousness in plants is indicated because they sense environmental changes and respond adaptively, integrating information for goal-directed behaviors, and making decisions along the way.	2. Goal-directed behavior is an evolutionary trait of all organisms and nonconscious processes, so adaptive responses alone do not prove consciousness.	**2. Interaction between Emotion and Cognition**: Functions sometimes considered cognitive are instead caused by a plant’s emotion, which does not have to be conscious. Emotion is necessary for complex information integration between cells, using structures like plasmodesmata and gap junctions instead of the brain.
3. Membrane potentials and electrical signals are similar in plants and animals, in ways that allow consciousness.	3. Electrical activity in plants is fundamentally different from animals (plants use H + and Cl− transport; animals use Na+ transport).	**3. Tissue Potentials and Signals**: Tissue potentials and electrical signals in plants enable **bodily emotion**, not necessarily consciousness. The complex tissue signaling system indicates plants may have a complex plant emotion system
4. Action potentials and other electrical signals for communication propagate, neuron-like, along phloem elements.	4. Phloem action potentials regulate osmosis, while variation potentials are too slow, infrequent, and variable to support the neuron-like signaling required for consciousness.	**4. Differences and Similarities between Neurons and Action Potentials**: Action potentials function like neurons combined with other mechanisms in plants, enabling the movement of electricity and energy, thus facilitating bodily emotions. Action potentials in plants have qualities that make it not possible for a plant to feel pain.
5. Plants, like animals with neurons, use electrical signals to integrate information for consciousness	5. Consciousness depends on recurrent electrical feedback for information integration, which plants lack since only forward signals are observed.	**5. Electrical Signals and Integration of Information**: Plants use electrical signals to integrate information into **emotional functions**. This emotional energy (*emotus plantarum*) is regulated through the frequency and functions of plasmodesmata.
6. Plants have a brain (“command center”) in the root.	6. The proposed “command center” consists of transient, undifferentiated cells lacking stable networks or synapses, making plant consciousness and memory impossible.	**6. Information Processing**: Plants do not have a single brain-like center, but process information through structures like plasmodesmata and gap junctions. Plant “cognition” is the unconscious processing of information carried by emotional energy into plant movements.
7. Plants show proactive, anticipatory behavior.	7. Plant behaviors are purely reactive to stimuli, whereas true proactive, goal-directed actions based on planning and mental maps—seen in some animals—have never been demonstrated in plants.	**7. Anticipatory Behavior**: The anticipatory behavior of plants is due to emotion, which is associated with anticipatory qualities in humans and animals.
8. Plants show classical associative learning, which indicates consciousness.	8. Classical learning in plants is unproven, and even if confirmed, it remains nonconscious and irrelevant to plant consciousness.	**8. Learning**: Unconscious emotion and its processing are sufficient for learning in plants
9. Plants communicate with each other in a purposeful manner and, hence, have conscious self-recognition.	9. Signal exchange and self–nonself recognition are basic biological adaptations, not evidence of consciousness or cognition.	**9. Communication**: Plant signaling is a complex communication system that has an emotional nature, rather than being a mechanical reflex or brain-like function. Plants communicate through emotional energy.
10. Detailed hypotheses, predictions and models can substitute for hard evidence of plant consciousness.	10. Proponents engage in excessive speculation, “piling theory upon theory far beyond the evidence”.	**10. Existing findings**: Research results suggesting plant sentience strongly indicate the existence of a plant **emotion system**, which can logically interpret existing findings
11. Plants show affective (emotional) consciousness.	11. The claim wrongly links classical learning to emotions, while true affective consciousness requires operant learning—absent in plants and even in nonconscious spinal cords.	**11. Emotus Plantarum and Affective Emotions**: Plant emotion is not affective because it does not involve feeling but is rather a more sensory and bodily emotion. The theory does not attribute psychic properties to plants.
12. Plants have image-based consciousness, based on internal representations.	12. There is no evidence for plant image-based consciousness, which requires internal environmental maps found only in certain animal nervous systems.	**12. Image-Based Consciousness vs. Emotion System**: Plants do not have image-based consciousness but possess an emotional system that resembles the human emotional system at the level of “core affect”.

Our new interpretation of the claims based on the emotus plantarum aim to bridge the current knowledge gap in plant emotions research. Emotus plantarum operates without the necessity of cellular consciousness and emphasizes that the emotional functioning does not require anything psychic or parapsychological. Emotus plantarum avoids the false dichotomy (mechanical vs. conscious) by proposing a third alternative: an unconscious emotional adaptive system. Furthermore, existence of emotus plantarum is proposed to be scientifically testable and measurable through physiological and biological measurements, such as analyzing electrical signals (cf. Volkov 2006; Fromm and Lautner 2007; Armada-Moreira et al. 2023), analyzing extracellular vesicles (cf. Cui et al. 2020; Karamanidou and Tsouknidas 2021; Ambrosone et al. 2023), and by studying the thermodynamics of plants. Next, the new scenario and reinterpretation of claims are outlined in more detail.

### Claim 1: Conscious cells and emotus plantarum

Some researchers defending plant sentience reason that conscious cells enable consciousness of plants (Mallatt et al. 2021; Baluška and Reber 2019). According to research regarding consciousness, description of consciousness cannot be complete without emotion (Candia-Rivera 2022). A recent review on plant sentience (Hansen 2024) highlights that some researchers of plant sentience have not considered plant emotion at all or have considered it as secondary to consciousness.

We think it is essential to consider emotion in the study of plant sentience, but not as affective consciousness. In our view emotions are more necessary to general biological processes than consciousness and it is more reasonable to begin the study of plant sentience from research of emotus plantarum. If plants exhibit unconscious emotional responses, it would then be more reasonable to investigate whether they possess sentience or forms of awareness.

Plant emotion theory does not require living cells to be conscious as separate cells. Unconscious emotional processes are possible. Such processes include many components of emotional processes, but not awareness (Delplanque and Sander 2021). Thus, such emotional processes do not require presence of conscious cells like many theories regarding plant sentience. Although plant behavior exhibits several components analogous to emotion, the possibility that plants may possess forms of emotion has often been underestimated or overlooked. This is largely because emotion is traditionally defined as requiring awareness, subjective feelings, or consciousness (cf. Struik 2023). However, emotions may exist without emotional experience and awareness of them as feelings.

If awareness is interpreted through the framework of Integrated Information Theory (Trewavas 2021), e*motus plantarum* may be understood as representing a form of plant-specific awareness (cf. Barlow 2015). This would position *emotus plantarum* as conceptually closer to the notion of “core affect.”

### Claim 2: Interaction between emotion and cognition

Emotion regulation is one type of cognitive function in humans that can happen automatically. At a fundamental level, the processing of emotional stimuli involves mechanisms characterized by coordinated neurohormonal responses that are activated in the presence of a stressor (Dolcos et al. 2011). In plants, stress signaling interacts with multiple endogenous signaling networks, including hormonal, electrical, and calcium-based pathways, in a coordinated manner that parallels fundamental aspects of emotional stimulus processing at a basic physiological level.

Signaling system of the plant functioning in connection with stress response signals, is according to our hypothesis a part of emotion system of the plant. We propose stress response functions in connection to emotus plantarum or are functions of emotus plantarum.

In humans, emotion serves as an essential component in the integration of information within highly connected neural networks, facilitating complex cognitive and emotional behaviors. Analogously, it is plausible that plants, too, may require emotion processes to enable complex connectivity and information integration across cellular networks. In our view emotion is necessary for such functions that however may function without consciousness. In plant instead of the brain, plasmodesmata and gap junctions functions in coalition with information flows (Koenig and Hoffmann-Benning 2020; Yatta 2023) that are carried by moving energy, which in our view is kinetic emotional energy, not mechanical.

### Claim 3: Tissue potentials and signals

The electrical signaling system in plants acts as a complex adaptive system to help plants adapt to their environment (Yatta 2023) and this system could be identified as the emotion system of plants. Emotions function as complex adaptive system in humans (Izard 2009). As evolutionary and behavioral adaptions are considered by several researchers indicating existence of emotions in case of humans and animals (Barrett 2012), it would be logical to assume same in the case of plants.

There are also oscillations in multiple aspects of plant growth. Oscillations make it possible for plants to respond to environmental fluctuations by adjusting their internal processes (Damineli et al. 2022). In our view also such behaviour and ability of plant indicate existence of bodily emotions, emotus plantarum, in plants.

The plant signaling system integrates multiple forms of communication, including rapid alterations in membrane potential driven by ion fluxes, as well as oscillations in membrane potential associated with growth processes. Some electrical signals are responsible of slow changes in cell potential over extended period of time while other signals are rapid. Some signaling is linked to leaf movements and other signaling is systemic. There is also signaling related to plant to plant communication and electrical reactions to environmental factors (Yatta 2023).

### Claim 4: Differences and similarities between neurons and action potential

Plant electrical signal data can be considered as electrophysiological phenotype typical to plants (Yatta 2023), that according to our view indicates emotus plantarum, not machine like or human like electrical signaling. We propose that there is kinetic emotional energy flowing in plants, not just mechanical energy. Plant action potentials, which function in a manner analogous to nerve impulses (Yatta 2023), enable the propagation of electrochemical energy throughout plant tissues, facilitating coordinated physiological responses, even though they do not replicate all functions of an animal-like nervous system.

### Claim 5: Electrical signals and integration of information

Brosch et al. (2013) distinguish between reflexes and emotions, noting that “a reflex or a fixed action pattern inflexibly links a certain stimulus to a response, whereas an emotional response creates a latency period during which physiological responses can be initiated and several appropriate action tendencies can be prepared while the situation is further analysed.” (Brosch et al. 2013, p. 1). Considering this distinction, certain plant responses, given the way plants integrate information and modulate physiological activity, appear to more closely resemble emotional responses than purely mechanical reflexes.

Because stress-responsive signals and the physiological state of the plant can modulate the propagation of other signaling pathways (Yatta 2023), plant responses are, in our view, more complex than simple reflexes and cannot be reliably predicted to produce a fixed outcome. This indicates that responsiveness of the plant is not just mechanical, but there exists also emotional aspect in plants that we propose to be called emotus plantarum.

When multiple stress conditions occur simultaneously, they generate distinct downstream signaling patterns that were once considered general responses, highlighting the complexity of plant stress integration (Lamers et al. 2020). Furthermore, stress-response and growth-regulation pathways interact reciprocally at multiple levels, indicating that plant responses involve a coordinated balance between environmental adaptation and developmental processes (Zhang and Zhao 2020).

It has been found that plants do have a system by which they react to stressors. This system is characterized by the coordinated interplay of multiple molecular classes, many of which are actively transported and function collectively to regulate plant signaling and responses (Koenig and Hoffmann-Benning 2020).

### Claim 6: Information processing

Emotional episodes function like an extension of biological processes in which natural organisms self-organize. From the point of view of entropy emotional systems function as affected by natural environmental conditions as a subprocess of dynamic living systems (Wells 2019). From this perspective, the information-processing mechanisms underlying the regulation of *emotus plantarum* in plants can be examined.

### Claim 7: Anticipatory behavior

According to Mallatt et al. (2021) plants do not exhibit anticipatory behavior. According to our view anticipatory behavior is common to all living biological beings including plants (Sims 2023). We argue that plants exhibit anticipatory behavior, but this does not necessarily indicate sentience. The anticipatory behavior of plants is due to emotion system. Emotion has been also associated with anticipatory qualities in humans and animals (Castelfranchi and Miceli 2011). According to Sims (2023, p. 130): “… intricacies of biological adaptive processes cannot be understated. The many paths to anticipatory behavior are no exception”.

### Claim 8: Learning

According to Tyng et al. (2017, p. 17): “All cognitive activity is motivated from ‘underneath’ by basic emotional and homeostatic needs (motivational drives) that explore environmental events for survival while facilitating secondary processes of learning and memory.” We propose that in plants, learning may not be possible without emotus plantarum, which functions to maintain homeostasis. In this view, learning and memory processes in plants could be considered secondary outcomes of emotus plantarum.

### Claim 9: Communication

Plant signaling is complex communication system through which plant communicates with the environment and between cells. In our view such communication is not mechanical or brain-like function but has emotional nature. Plant communication systems are so multiform and complex that in our view it cannot be just mechanical reaction or an automatic involuntary response. According to plant emotion scenario plants communicate through emotus plantarum that responds to sensations and carry information by moving energy.

According to study of Witzany (2006, p. 169): ”Almost without exception, plant communication are parallel processes on multiple levels, (A) between plants and microorganisms, fungi, insects and other animals, (B) between different plant species as well as between members of the same plant species; (C), between cells and in cells of the plant organism.

Plants sensitively respond to sounds that impact behavior of plants (Jung et al. 2018; Demey et al. 2023). In our view sensitivity of plants to different sounds indicates emotional behavior of the plant. According to previous research, electrical reactivity of the plant is measurable in presence of movements of human body (De La Cal et al. 2023). Current research has not identified the mechanisms underlying plant sensitivity to human movement in their environment (De La Cal et al. 2023). We propose that this sensitivity may be mediated by the plant’s *emotus plantarum*, a system whose multiple functions contribute to the integration of environmental cues and coordinated physiological responses.

### Claim 10: Existing findings

We propose an alternative framework for investigating plant intelligence, sentience, and consciousness, beginning with the consideration of emotion in plants from a non-neurocentric perspective. By establishing a foundation based on plant-specific emotional processes, research on plant intelligence can be approached from a novel angle, potentially allowing studies of plant sentience to progress without becoming constrained by traditional neurocentric assumptions (Lee and Calvo 2023; cf. Hansen 2024). Research on plant consciousness, sentience, or awareness could begin by investigating whether plants possess the necessary components for such qualities, such as emotion, or whether they are capable of processing specific patterns or frequencies of signaling required to support conscious-like states (cf. Baluska and Souza 2024).

Researchers in plant cognition have suggested that plants are capable of higher-level cognitive processes that go beyond simple mechanical responses to stimuli. For instance, plants can exhibit adaptive decision-making when confronted with challenging conditions. These findings indicate that plants may display attention-like states, selectively responding to multiple simultaneous environmental cues (cf. Baluska and Souza 2024). We hypothesize that this plant “attention” may be a function of *emotus plantarum*, or plant-specific emotional processes, which may operate independently of the neural oscillations typically associated with attention in humans.

In our view, the majority of existing research on plant sentience to date provides stronger evidence for the presence of a plant emotion system rather than for full consciousness, sentience, or necessarily awareness. Findings from multiple studies can be coherently interpreted as supporting the existence of plant-specific emotional processes. By adopting this slightly revised interpretive framework, the current impasse in research on plant cognitive qualities can be overcome, allowing the field to advance in a logically consistent manner along several investigative directions.

Concept emotus plantarum makes it possible to study plant sentience without changing meaning of cognition as it is generally understood. Miguel-Tomé and Llinás (2021) propose broadening the concept of nervous system so that the evolution of plants can be better studied. Instead of broadening the concept of nervous system, we propose the introduction of new concept to plant science: emotus plantarum.

To accommodate fundamental differences in relation to animal and human emotional systems, the introduction of a distinct conceptual framework is necessary. Within the proposed concept of emotus plantarum, neural or “brain-dependent” components of emotional functioning are excluded, thereby isolating and retaining only those functional aspects of emotion that do not require are centralized nervous system.

Changing existing established concepts might unnecessarily cause confusion in other fields of research and in the debate on plant consciousness. Emotus plantarum, plant specific emotion, is a concept that can be added to the discussion without the need to alter existing scientific concepts. Many researchers have noted that several plant functions are highly species-specific. Considering this, a more plant-specific conceptual term can be useful to improve conceptual clarity.

### Claim 11: Emotus plantarum and affective emotions

Research on plant sentience and intelligence connect plants to “affective consciousness” (Mallatt et al. 2021). Emotus plantarum is neither “affective” or “consciousness”. Emotus plantarum does not involve feeling but is more sensory and bodily emotion without conscious emotional experience. Theory regarding emotus plantarum does not attribute psychic properties to plants. The criticism of affective (emotional) consciousness of plants is based on two assumptions concerning which organisms “possess consciousness” (Mallatt et al. 2021), which neither apply to emotus plantarum.

In humans, neurotransmitters function as key components of both the nervous system and the neural mechanisms underlying emotions. According to Wang et al. (2020), emotional states arise, in part, from the activity of neurotransmitters. Neuromodulators such as dopamine, serotonin, and norepinephrine play key roles in modulating emotional states in humans (Lindquist et al. 2012; Lövheim 2012; Wang et al. 2020). Given that emotions in humans are associated with the activity of neurotransmitters, and that plants produce neurotransmitters including dopamine, serotonin, and norepinephrine, it is possible to conceptualize a plant emotion signaling system, distinct from a nervous system.

Furthermore, several studies have emphasized the role of plant action potentials, which do not require the presence of neurons, in facilitating complex physiological and behavioral functions in plants (Awan et al. 2019; Robinson and Draguhn 2021; Lee and Calvo 2023). We propose that plant action potentials may contribute to affective-like processes in plants. Although these electrical signals neither involve neurons nor instantiate brain-like computational functions, they can mediate the propagation of energy and information and coordinate physiological and behavioral responses, thereby functionally substituting for certain roles typically attributed to neuronal mechanisms.

### Claim 12: Image-based consciousness vs. emotion system

According to the theoretical framework of *emotus plantarum*, plants do not possess image-based consciousness or the capacity for imagination. Instead, they are thought to operate through a plant-specific emotional system, which mediates physiological and behavioral responses without relying on the representational or imaginative processes characteristic of animal consciousness. This suggests that plant cognition and responsiveness may be fundamentally grounded in emotion system regulatory mechanisms rather than perceptual or imagery-based awareness.

### Limitations

This study offers a novel conceptual framework for understanding plant emotion through the lens of emotus plantarum, which provides a foundation for future research despite several inherent limitations. First, the scoping study primarily relied on articles indexed in the Scopus database using a limited set of search terms related to plant emotions and feelings. Expanding the search to include terms such as plant intelligence, plant consciousness, plant sentience, and plant thinking could identify additional relevant studies. To address potential gaps, the review was supplemented with highly cited works identified through backward referencing, helping to capture influential research beyond the initial search. Future studies could build on this approach and also conduct a systematic literature review to provide a more comprehensive synthesis of existing research.

Second, while there are currently no experimental studies directly testing the emotus plantarum framework, recognizing this concept opens opportunities to design empirical studies that could enhance our understanding of plant biology, behavior, and adaptive capacities.

Finally, questions remain regarding whether emotus plantarum might reflect a form of plant-specific, non–brain-based awareness, as well as the broader relationships among biological systems, emotion processes, and consciousness. These open questions present opportunities for interdisciplinary research that can deepen both philosophical and empirical insights. By highlighting these avenues, this review provides a constructive starting point for advancing the scientific study of plant emotion-like phenomena.

## Conclusions

The present analysis supports the hypothesis that plants possess an organized, biologically grounded emotion system termed *emotus plantarum* without invoking consciousness, sentience, or cognition in the human or animal sense. While some researchers have proposed that conscious cells enable plant consciousness, current evidence suggests that plants lack the structural and functional prerequisites for conscious awareness, including brain-like centers, recurrent electrical feedback, or synapses. Instead, plant behavior can be more parsimoniously explained by unconscious emotional processes that integrate environmental signals into adaptive responses.

Plant emotion, as conceptualized here, resembles the core affective components of human emotions but operates without awareness or psychic experience. It integrates multiple signaling modalities such as electrical potentials, phloem-mediated energy flows, plasmodesmatal communication, and chemical signaling—to coordinate adaptive movements and responses to environmental stimuli. These processes are complex, yet they function automatically, akin to bodily emotional responses in humans, rather than abstract cognitive processes. Accordingly, plant behaviors previously interpreted as cognitive, such as anticipatory responses, decision-making, and learning, are better understood as manifestations of this unconscious emotional system.

Analogous to the integration of emotion and cognition in humans, where neural hubs coordinate information flow across highly connected regions, plants achieve complex adaptive responses through dynamic interactions between signaling pathways. Phloem networks, plasmodesmata, and gap junctions facilitate the transmission and integration of emotional energy, enabling orchestrated movements and stress responses. For instance, reciprocal regulation between growth and stress-response pathways demonstrates functional parallels to emotion-based regulation in animals, guiding plant development under environmental constraints. Similarly, the modulation of signal speed, direction, and intensity reflects a sophisticated, context-dependent coordination more consistent with emotional processing than with reflexive or purely mechanical reactions.

The concept of *emotus plantarum* also provides a framework for studying plant signaling and behavior in a measurable, scientific manner. Electrical signals, energy flows, and chemical mediators can be investigated using established techniques such as electrophysiology, microelectrode recording, ion-selective electrodes, confocal microscopy, transcriptomics, proteomics, and computational modeling. These approaches allow for quantifiable, testable exploration of plant emotional processes without resorting to biopsychism or parapsychological explanations.

Importantly, the emotus plantarum concept reconciles divergent perspectives in plant research by accounting for both evidences suggesting plant sentience as well as counterarguments against it. It provides a species-specific framework that explains the sensitivity, adaptability, and complex behaviors of plants without attributing consciousness or feelings to them. By focusing on the biologically and physically measurable aspects of plant emotional systems, *emotus plantarum* clarifies the mechanisms underlying plant responses, guiding future research and opening new directions for the study of intercellular signaling, adaptive behavior, and evolutionary trajectories of emotion across different groups of organisms.

In summary, plants exhibit a sophisticated, unconscious emotion system that orchestrates perception, signaling, and adaptive behavior. This system parallels human bodily emotions in its integrative and regulatory functions but operates independently of consciousness. Recognizing *emotus plantarum* as a scientifically tractable concept advances our understanding of plant biology, bridging gaps between perception, signaling, and adaptive behavior, while maintaining adherence to empirical evidence and avoiding anthropomorphic interpretations.

## Data Availability

No datasets were generated or analysed during the current study.
